# Luteoma of Pregnancy Presenting with Severe Maternal Virilisation: A Case Report

**DOI:** 10.1155/2016/3523760

**Published:** 2016-08-31

**Authors:** Vincenzo Rapisarda, Francesco Pedalino, Veronica Concetta Santonocito, Giorgia Cavalli, Giuseppe Zarbo

**Affiliations:** ^1^Obstetrics and Gynecology Department, Ospedale Vittorio Emanuele-Policlinico-S. Bambino, 95123 Catania, Italy; ^2^Obstetrics and Gynecology Department, ASST Papa Giovanni XXIII, 24127 Bergamo, Italy

## Abstract

Luteoma of pregnancy is a rare, benign condition characterized by a tumor-like mass of the ovary that emerges during pregnancy and regresses spontaneously after delivery. It is usually asymptomatic and the diagnosis is generally incidental. Luteoma arises from the proliferation of luteinised cell under the influence of *β*-hCG and can be hormonally active, with production of androgens resulting in maternal and fetal hirsutism and virilisation. We report a case of a 25-year-old primigravida who presented at 28 weeks of gestation with virilisation symptoms. Serum androgen levels were seven-hundred-fold higher than normal. A diagnosis of pregnancy luteoma was made at the time of caesarean section. The ovarian mass, serum androgen levels, and the condition of the patient improved after delivery.

## 1. Introduction

Pregnancy luteoma is a rare tumor-like lesion of the ovary, first described in 1963 by Dr. Sternberg, that occurs during pregnancy and generally resolves completely after delivery [1].

It is a benign condition: only in 30% of cases the mass is hormonally active leading to secretion of androgens causing masculinization in mother and female babies [2, 3].

To date, fewer than 200 cases have been reported in literature and most of the patients were mildly symptomatic [2, 4].

An accurate diagnosis is fundamental for the management of the mother and fetus since it can be confused with ovarian malignancy [5].

We report the case of a Caucasian woman with virilisation worsening symptoms in the second trimester of pregnancy.

## 2. Case Presentation

A 25-year-old primigravida presented at 28 weeks of gestation with symptoms of facial acne and abdominal and facial hirsutism appearing first at 23 weeks of gestation.

**Figure 1:**
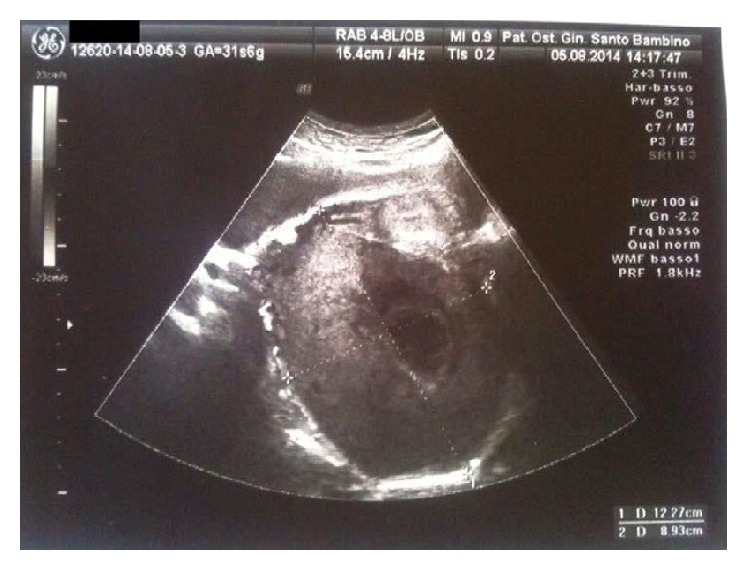


Ultrasound imaging showed a viable fetus with a normal growth and morphology and a 12 × 9 cm right adnexal mass with necrotic areas and low color Doppler signals (Figure 1). The left adnexa was unremarkable. The blood test showed a mild elevated level of transaminases, negative tumoral markers, and elevated serum level of testosterone (6270 ng/dL; normal range 0.4–4.1 ng/dL), SHBG (689,6 nmol/L; normal range 18–114 nmol/L), androstenedione (≤200 ng/dL; normal range 0.4–4.1), and DHEAS (645,9 *μ*g/dL; normal range 98,8–340). A magnetic resonance was proposed but patient declined the study.

In the following weeks blood tests and ultrasound follow-up showed progressive worsening of the clinical situation with an aggravation of hirsutism (Ferriman and Gallwey score: 18) and the onset of clitoromegaly and deepening voice.

A caesarean section was performed at 34 weeks of gestation because of the worsening of laboratory tests and symptoms (with also the appearance of severe right hydronephrosis), the incomplete diagnosis due to the patient's denied consent, and the suspected ovarian malignancy. Antenatal corticosteroids with two doses of betamethasone (12 mg i.m.) were administrated to the patient prior to surgery.

**Figure 2:**
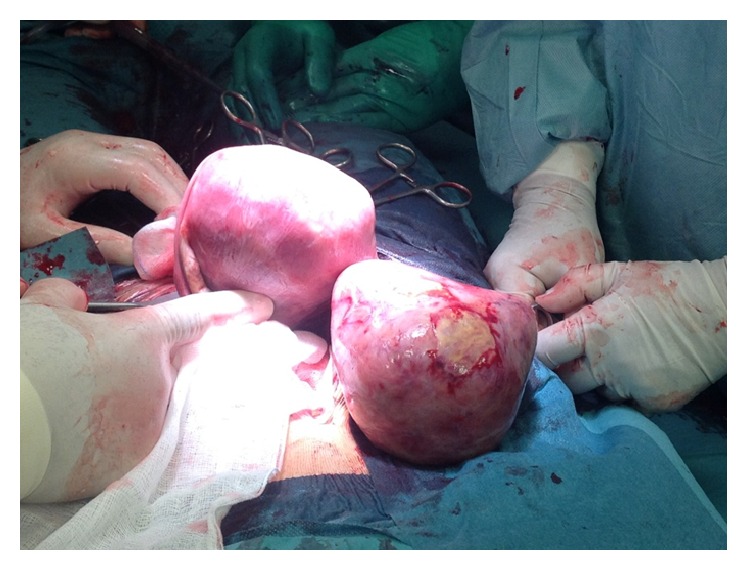


During the operation the uterus and left ovary were macroscopically normal, while an extremely enlarged right ovary mass was observed (Figure 2); unilateral oophorectomy was then performed. Final pathologic examination revealed pregnancy luteoma.

A healthy male infant of 1560 grams was delivered; Apgar score was 8 at the first minute and 10 at the fifth minute.

A blood sample of umbilical cord showed elevated levels of testosterone and DHEAS (200 ng/dL and 201 *μ*g/dL, resp.).

After the resection, the serum androgen levels gradually fell down as shown in Table 1.

Postoperative course was uneventful and the patient was discharged 4 days after the caesarean section.

**Figure 3:**
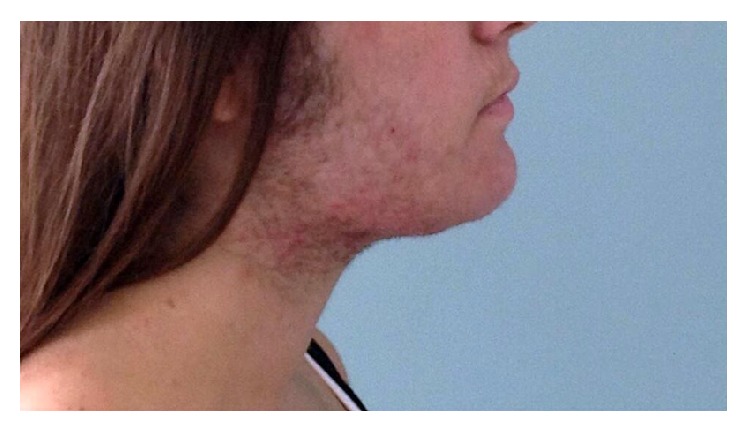
Patient at 23 weeks of gestation.

**Figure 4:**
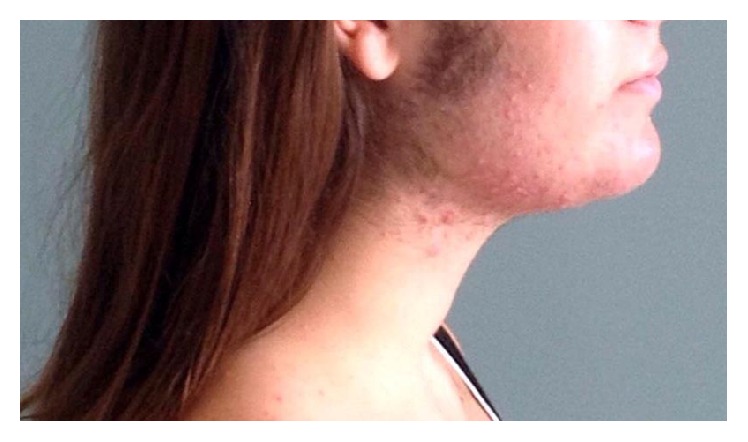
Four months after delivery.

**Figure 5:**
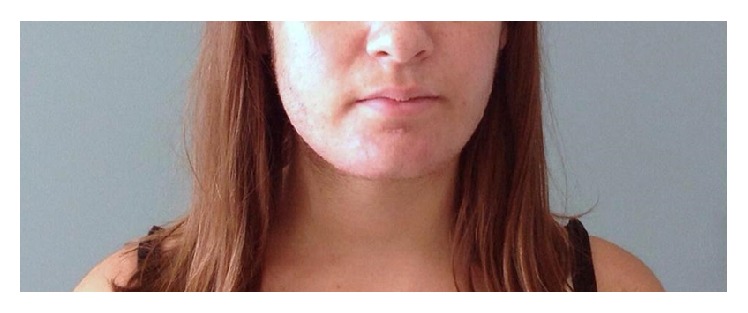
Six months after delivery.

Blood samples at 30, 60, and 120 days from delivery showed regular androgen levels with a progressive improvement of virilisation symptoms (Figures 3, 4, and 5).

## 3. Discussion

Pregnancy luteoma is a nonneoplastic lesion of the ovary occurring during pregnancy that is commonly reported in Afro-Caribbean women generally in the third or fourth decade of life and in those with preexisting PCOS [6].

The exact incidence is unknown because most of the patients are asymptomatic and the diagnosis is generally made incidentally during a caesarean section or a postpartum tubal ligation.

The aetiology of pregnancy luteoma is unclear: it is thought that it arises from the proliferation of luteinised cell under the influence of *β*-hCG [2]. Macroscopically it appears as solid brown-yellow masses with variable size up to 20 cm of diameter and with haemorrhagic spots. Only in one-third of the cases the mass is bilateral. Microscopically the nodules are made up of cells arranged in trabecular or follicular pattern with stromal cell proliferation [7].

Approximately 25% of women with pregnancy luteoma have androgen hypersecretion and among them 10–50% present clinical signs of hyperandrogenism such as hirsutism in androgen-dependent areas of predilection (such as chin, upper lip, linea alba, and chest), acne on the face, shoulders, back, and chest, hair loss, and masculine symptoms such as clitoromegaly and deepening voice. Some symptoms such as acne and hair loss are fully reversible but hirsutism, deepening voice, and clitoromegaly may be only partially reversible after delivery.

Up to 60–70% of female fetus may be affected with different degree by virilisation (hirsutism, clitoromegaly, and genitalia malformation) [2, 3]. The risk of virilisation of the fetus depends on the gestational period of the beginning of the hyperandrogenism (higher in the first trimester of pregnancy) and on the placental aromatase functionality. Male fetus is not affected by these alterations but some authors suggest that the intrauterine exposure to elevated androgens levels may lead to an increased risk of mental retardation and hypogonadism.

Rarely pregnancy luteoma of large size may cause ovarian torsion or tumor rupture with symptoms of acute abdomen, haemoperitoneum, or compression of the pelvic structures such as ureters [6].

Diagnosis and follow-up of this condition should be made with noninvasive methods; at ultrasound scan luteoma appears as a solid, single, or multinodular mass. It may be unilateral or rarely bilateral with a cystic appearance due to the presence of haemorrhagic foci. However, because ultrasound exam during the second half of pregnancy may be challenging, considering the poor image quality caused by the uterine enlarged volume, magnetic resonance may be useful in the diagnostic evaluation of the patient [2].

Differential diagnosis must be made with other solid ovarian neoplasm such as luteinised thecoma, granulosa cell tumor, or Leydig cell tumor [7].

The management of pregnancy luteoma depends upon the symptoms and personal condition; different authors reported that surgical treatment with unilateral salpingo-oophorectomy was the most frequent option; however, whenever possible, conservative treatment should be recommended considering the benign nature of the lesion [3, 8]. In fact, classically, luteomas resolve by 3 months postpartum; androgens levels reduce in the first 2-3 weeks and clinical virilisation symptoms generally disappear in 2–6 months after delivery. In our case salpingo-oophorectomy was performed because of the impossibility of making a complete antenatal evaluation of the patient and in consideration of the mass volume.

Recurrence of luteoma in subsequent pregnancies is possible but rare.

## 4. Conclusion

Pregnancy luteoma represents a benign pregnancy related condition that generally resolves spontaneously after delivery. Antenatal accurate diagnosis is challenging but extremely important in order to optimize the obstetrical management of the patient and to improve the maternal and fetal outcome.

## Figures and Tables

**Table 1:** Androgen levels after delivery.

	60 minutes after surgery	24 hours after surgery	48 hours after surgery
Testosterone (ng/dL) (normal range 6–82)	2740	353	147
DHEAS (*μ*g/dL) (normal range 98.8–340)	535.5	203.5	146
S-Delta-4-androstenedione (ng/mL) (normal range 0.4–4.1)	140	8.6	5.4
S-SHBG (nmol/L) (normal range 18.0–114.0)	538.8	444.1	446.7
